# Rate of first recorded diagnosis of autism and other pervasive developmental disorders in United Kingdom general practice, 1988 to 2001

**DOI:** 10.1186/1741-7015-2-39

**Published:** 2004-11-09

**Authors:** Liam Smeeth, Claire Cook, Professor Eric Fombonne, Lisa Heavey, Laura C Rodrigues, Peter G Smith, Andrew J Hall

**Affiliations:** 1 Department of Epidemiology and Population Health, London School of Hygiene and Tropical Medicine, Keppel Street, London WC1E 7HT, UK; 2 Department of Infectious and Tropical Diseases, London School of Hygiene and Tropical Medicine, Keppel Street, London WC1E 7HT, UK; 3 Department of Psychiatry, McGill University, Montreal, Canada; 4 Institute of Psychiatry, Kings College, London, UK

## Abstract

**Background:**

There has been concern that the incidence of autism and other pervasive developmental disorders (PDDs) is increasing. Previous studies have been smaller, restricted to autism (excluding other pervasive developmental disorders such as Asperger's syndrome), included boys only, or have not been based on a national sample. We investigated time trends in the rates of diagnosis of pervasive developmental disorders.

**Methods:**

We analysed the rates of first diagnosis of pervasive developmental disorders among people registered with a practice contributing to the United Kingdom General Practice Research Database during the period 1988 to 2001. We included 1410 cases from over 14 million person-years of observation. The main outcome measures were rates of diagnosis of pervasive developmental disorders by year of diagnosis, year of birth, gender and geographical region.

**Results:**

The rate increased progressively from 0.40/10,000 person-years (95% CI 0.30 to 0.54) in 1991 to 2.98/10,000 (95% CI 2.56 to 3.47) in 2001. A similar change occurred in the age standardised incidence ratios, from 35 (95% CI: 26–47) in 1991 to 365 (95% CI: 314–425) in 2001. The temporal increase was not limited to children born during specific years nor to children diagnosed in a specific time period. The rate of diagnosis of PDDs other than autism rose from zero for the period 1988–1992 to 1.06/10,000 person-years in 2001. The rate of diagnosis of autism also increased but to a lesser extent. There was marked geographical variation in rates, with standardised incidence ratios varying from 66 for Wales to 141 for the South East of England.

**Conclusions:**

Better ascertainment of diagnosis is likely to have contributed to the observed temporal increase in rates of diagnosis of PDD, but we cannot exclude a real increase.

## Background

The term pervasive developmental disorder (PDD) refers to a range of disorders with onset in childhood characterised by abnormalities in the domains of language development, communication abilities and social interactions and by a rigid, repetitive pattern of behaviours and interests [1]. Within PDDs, autism refers to children meeting full diagnostic criteria for the above three domains of developmental impairments and onset before the age of three years. The other PDDs are Asperger's syndrome (a less severe form of PDD with the features described above but without language delay and with intelligence within the normal range), pervasive developmental disorder not otherwise specified (not fulfilling complete criteria for autism), and childhood disintegrative disorder (a severe form of autism developing in previously normal children who undergo massive regression between the ages of 2 and 10 years) [2]. Rett's disorder (occurring only in girls, characterised by acquired microcephaly, regression and neurological signs) is also classified as a PDD in ICD-10 but has a known genetic aetiology [3] and is not included in the incidence estimates presented in this paper.

There have been a number of reports that the incidence of PDD is increasing [3] and the prevalence for all PDDs appears to be around the 60/10,000 in recent studies in the United Kingdom [4] and the United States [5]. There is some evidence that increased prevalence rates are due to a broadening of the concept of PDD and changes in diagnostic practice [6,7]. This has not gone unchallenged, however, and it is likely that increased awareness and changes in educational and social policies account for some of the recent upward trend in rates[6,8]. However, a real increase in the incidence cannot be ruled out [3].

As part of a case-control study to identify risk factors for autism and other PDDs [9], we have investigated time trends in the incidence of the diagnosis of PDDs using data obtained from the United Kingdom General Practice Research Database (GPRD).

## Methods

### The General Practice Research Database

The GPRD was set up in 1987, then known as the VAMP (Value Added Medical Products) Research Bank [10]. It consists of the electronic clinical records of patients registered with contributing general practices and aims to include complete prescribing and diagnostic information. The practices included in the GPRD are broadly representative of all practices in England and Wales in terms of geographical distribution, practice sizes and the age and gender distributions of registered patients [11]. Contributing practices must meet a range of data quality criteria before they are included in the GPRD. The quality of the information in the database, including the completeness of recording of diagnoses made in medical facilities outside the practice, has been validated in a number of independent studies and has been found to be high [10]. There is excellent agreement between prescribing data from the GPRD and national data from the Prescription Pricing Authority [12]. General practices contributing to the GPRD originally used a software system called VAMP but during the late 1990s most practices switched to a new software system called VISION. At the start of the study period, general practitioners contributing to the GPRD used a modified version of the Oxford Medical Information Systems coding system (OXMIS) to record diagnoses [13], but in later years most practices used the READ coding system [14].

Each individual is assigned a unique identification number and all data that could identify individuals are removed from records before they are incorporated into the GPRD.

#### Study population and denominators

The information analysed in the present study comprised the electronic medical records of children born in 1973 or later and registered with general practices contributing data to the GPRD during the period 1^st ^January 1988 to the 31^st ^December 2001. Such children will have been eligible at some time in their life for MMR vaccination, an exposure of particular interest in the case-control study [9].

For a specific general practice, the start of the observation period for this study was taken as the later of either the 1^st ^January 1988 or the date at which the practice started contributing data to the GPRD. The end of the observation period was the date at which the practice stopped contributing data to the GPRD, or the 31^st ^December 2001 if this was earlier. Individual patients were included in the study during the times within the observation period that they were registered with a practice contributing to the GPRD. The number of practices included in the GPRD varied during the study period, rising from 34 in 1988 to 557 in 1996 then falling to 380 by 2001.

Age- and gender-specific rates were calculated for people born in 1973 or later and registered with contributing general practices during each calendar year. Those registered for part of a particular year contributed time at risk for the period they were registered. People were classified by geographical location of their general practice by country for Wales, Northern Ireland and Scotland, and by the eight former administrative National Health Service regions for England.

### Identification of cases

When a patient first registers with a practice contributing to the GPRD, past medical events and diagnoses judged to be clinically important by the general practitioner are recorded in the electronic record, with the date of each diagnosis where available. While a patient is registered with a contributing practice, all new diagnoses and all new drug therapies or changes in existing therapies are recorded contemporaneously. Cases were defined as people born in 1973 or later who had a first diagnosis of PDD entered into their general practice record while registered with a practice contributing to the GPRD during the study period. Cases were identified by searching the complete medical record of all people registered with participating practices during the study period for either OXMIS or READ clinical codes indicating a diagnosis of PDD: Appendix [see Additional file 1]. People with a diagnosis of a PDD made prior to their observation period, and people whose PDD diagnosis was the first entry recorded when they joined a practice, were classified as prevalent cases and were excluded from the incidence estimates.

On the basis of the recorded diagnoses patients were classified as 'autism' or 'other PDD'. Those with autistic disorders and similar presentations were grouped in the autism category and other descriptions (such as Asperger's syndrome) were grouped in the other PDD category.

#### Analysis

Crude rates and age standardised incidence ratios for first diagnosis of all PDDs, autism, and other PDDs were calculated by year of diagnosis and gender and for different geographical regions. Because of low numbers of cases in some years, indirect standardisation was used with rates during the whole study period as the standard rates. The age-standardised incidence ratios presented take into account variations in the age structure of the population in different time periods and different geographical regions. Rates of first diagnosis were calculated by year of diagnosis and year of birth, both in two year intervals.

### Ethical approval

Ethical approval for the study was obtained from the Scientific and Ethical Advisory Group of the GPRD and from the ethics committee of the London School of Hygiene and Tropical Medicine.

## Results

We identified 1410 persons with a first recorded diagnosis of PDD during the study period: 1097 were categorised as autism and 313 as other PDD, for 294 (94%) of whom the diagnosis was Asperger's syndrome (appendix).

Table 1 shows the crude rates of first diagnosis of PDD and age standardised incidence ratios by year of diagnosis and gender.

The person-years at risk varied from year to year because of changes in the number of contributing practices, which increased until the mid 1990s and then fell. During the study period incidence rates increased progressively for both males and females. The patterns of crude rates and of standardised incidence ratios were very similar. The overall rate increased seven-fold from 0.40/10,000 person-years (95% CI 0.30 to 0.54) in 1991 to 2.98/10,000 person-years (95% CI 2.56 to 3.47) in 2001. A similar change occurred in the age standardised incidence ratios, from 35 (95% CI: 26–47) in 1991 to 365 (95% CI: 314–425) in 2001. The overall male to female ratio was 4.8 and during 1991 to 2001 ranged from 2.7 to 8.4 (chi-squared test for heterogeneity, p = 0.21), with no clear temporal trend.

Table 2 shows the number of cases, person-years of follow-up and rate of first recorded diagnosis by date of diagnosis and by date of birth, both in two year intervals. The diagonally linked numbers identify rates among children of the same age in different years. The temporal increase observed in rates of diagnosis is not limited to children born during specific years or to children diagnosed in a specific time period. The rates of diagnosis for children at the same age, born in successive birth cohorts, increased in each successive cohort, including for relatively old ages at diagnosis. For example, for diagnoses at the ages of two to four years, the rates of new diagnosis per 10 000 person-years from 1992–93 to 2000–01 were 1.41, 2.70, 3.07, 5.56 and 7.74. Corresponding rates at ages eight to ten years were 0.36, 0.40, 0.67, 2.46 and 4.46.

Table 3 shows the rates of first recorded diagnosis and age standardised incidence ratios of autism and other PDDs by year of diagnosis.

There was a striking rise in the rate of diagnoses of other PDDs: from zero for the period 1988 to 1992 rising to 1.06/10,000 person-years by 2001. Over the study period the rate of diagnosis of autism also increased substantially. The patterns were broadly similar for males and females (data not shown). Few diagnoses of other PDDs were made before 1997, but by 2001 over one third of all diagnoses were PDDs other than autism.

Table 4 shows the rates of first recorded autism and other PDDs by geographical area.

There was marked geographical variation in rates of first diagnosis, with the lowest rate in Wales being less than half the rate in the South East of England (chi-squared test for heterogeneity in SIRs by region, p < 0.001). In general, the regional variations were less marked for autism than for other PDDs and the ratio of the diagnoses of autism compared with other PDDs varied greatly between areas (chi-squared test for heterogeneity, p = 0.0006).

## Discussion

There was around a 10-fold increase in the rate of first recorded diagnoses of PDDs in United Kingdom general practice medical records from 1988–92 to 2000–01 (table 1). The increase was more marked for PDDs other than autism but the increase in autism was also striking (table 3). If these changes indicate a true increase in the incidence of the conditions it is of great public health importance. However, it is probable that the increase is due, at least in part, to changes in the ascertainment and diagnosis of the conditions.

### Factors that could have affected the results

Some of the general practices contributing data to the GPRD will provide anonymised copies of hospital letters and specialist reports on individual patients. Of patients with a recorded diagnosis of PDD in the GPRD, including prevalent cases when first registered, 446 were registered with 203 general practices willing to provide this service. For 80 of these, medical records were not available as the patient was no longer registered with the general practitioner. We obtained complete case records including copies of hospital clinic letters and specialist reports for 318 (87%) of the remaining 366 people. These were reviewed by a psychologist (LH) and a random sample of 50 records were also reviewed by a child psychiatrist (EF), both of whom have long experience in the field of autism. They judged that a PDD was likely to be present in 294 children (92.5%)[15]. For the 211 patients (of 318) who were first diagnosed with a PDD after they entered the GPRD (and thus are included in this paper), a diagnosis of PDD was confirmed for 193 (91.5%). Thus the positive predictive value of a recorded diagnosis of PDD in the electronic record was high, but we were not able to assess the sensitivity of the ascertainment of cases, that is how often the diagnosis of a PDD may have been missed or not recorded.

Of the 211 cases reviewed and included in this paper, 5 (2.4%) were classified as 'other-PDD' on the basis of their electronic record only, whereas in the validation, 32 (15.1%) met diagnostic criteria for a PDD other than autism. Thus it is likely that a proportion of people in the 'autism' diagnostic category in this paper have a form of PDD other than autism. The inaccuracy of diagnostic descriptions of different PDDs within the GPRD is likely to reflect changes in the definition of PDD over the past two decades, in particular a broadening of PDDs other than autism [1,16,17]. Many children with Asperger's syndrome or pervasive developmental disorder not otherwise specified would only have been assigned a diagnosis of PDD from the latter half of the 1990s. In the earlier period such children may either not have received a diagnosis of PDD at all or have been diagnosed as autism. Inflation in the number of cases in later years could have occurred as other PDD diagnoses came into widespread use and some previously undiagnosed children were diagnosed. For example the OXMIS coding dictionary, used by most general practitioners contributing to the GPRD until the mid-1990s, has only two possible codes for autism and no clinical code for Asperger's syndrome, and this diagnosis could only be assigned when practices started to use the READ coding system. These changes explain the low level of diagnoses of other PDDs until the mid 1990s (table 3). During the study period there is likely to have been an increase in the diagnosis of high-functioning autism, as professionals have become more aware that autism can occur in people of normal intelligence [3]. Greater ascertainment of high-functioning autism may partly explain the increased incidence of autism as well as in the other-PDD diagnoses. That there was also a marked increase in the rate of diagnosis of severe disorders, however, suggests that better detection of less severe cases alone can not explain all of the increase. Two previous studies have demonstrated falls in the rates of diagnosis of mental retardation [6] and of non-specific developmental disorders [18] during the 1990s as the rate of diagnosis of autism increased. These patterns could be partly due to improvements in the detection and diagnosis of autism.

In the late 1980s and early 1990s autism was only likely to be diagnosed by specialist child psychiatrists who increased in number by 40% between 1988 and 2001 (personal communication: Royal College of Psychiatrists). Through the 1990s developmental and community paediatricians began to diagnose PDDs, and this, combined with increased awareness of autism and PDDs among the general public, may have contributed to increased ascertainment of the disorders [19]. The marked geographical variation in rates of diagnosis and in the ratio of diagnoses of autism to other PDDs (table 4) may reflect differences in service provision and parental awareness in different regions.

It is likely that children with PDDs other than autism will generally be first diagnosed at later ages than children with autism. Greater ascertainment of other PDDs during the latter part of the study period (table 3) could have led to the observed increase in incidence being largely restricted to older age groups. However, the increased rates were observed for all age groups (table 2).

Because of low numbers of cases in some years, indirect standardisation was used to calculate SIRs. When comparing SIRs, marked differences in the age distribution of the populations being compared can lead to a biased comparison [20]. However, the differences in age distributions were not great in our study and the patterns seen in the crude rates and the age standardised rates did not differ materially, suggesting the comparison of SIRs was valid.

The accuracy of the denominator data may have changed during the study period. When patients move geographical area they may delay registering with a new general practice until they have a specific reason to visit them. Thus our estimates of person-years at risk may be too low for those moving into a practice and too high for those moving out. Person-years at risk may also be inflated as patients who emigrate may omit to inform their general practitioner and, furthermore, administrative delays and errors may result in individuals being registered with more than one general practitioner. For example, in 1997 in England 51 million people were registered with a general practitioner [21] whereas the total population size was only around 49 million people[22]. It has also been suggested that in recent years the period of time for which patients who have moved out of an area remain registered with a general practice in that area has shortened as health authorities and general practices have streamlined procedures [23]. This may mean that the inflation of denominators could have been greater in the early years compared to later years. However, these factors could explain only a very small part of the increased rates observed. An additional issue is that the person-years used as denominators in our analysis did not exclude the period following diagnosis. However, given the relative rarity of the disease, this will have produced only a very small inflation in the denominators.

### Comparison with other studies

A previous study based on the GPRD assessed time trends in the diagnosis of autism from 1988 to 1999 [24,25]. The study did not examine other PDDs, was restricted to children aged less than 13 years and included a total of 305 cases. The previous paper was based on a sub-set of the GPRD data held by the Boston Collaborative Drug Surveillance Program. This is reflected in the person-years of observation contributing to the two studies – just under 3.1 million person years in the previous study compared with over 14 million person years in our paper. The two studies were undertaken in substantially different populations. However, the extent of the rise in incidence in the two studies was similar: from around 0.3/10,000 person-years in 1989/1990 to around 2.0/10,000 person-years by 1999 [24]. In addition, the increased risks of autism observed in successive birth cohorts were similar to those observed in our study [25]. A study in the West Midlands area of the United Kingdom based on diagnoses made at child development centres found cumulative incidence rates of autism for children between the ages of one to four years of 2.22/10,000 person-years in 1991–3 (based on 20 cases), rising to 4.75/10,000 person-years in 1994–6 (based on 42 cases) [26]. The corresponding cumulative rates from our study were 1.00/10,000 person-years in 1991–3 rising to 1.97/10,000 person-years for 1994–6. Restricting our data to the West Midlands region we observed rates of recorded diagnosis of autism of 1.57/10,000 person-years in 1991–3 and 1.56/10,000 person-years in 1994–6, based on 14 and 16 cases respectively. It is unclear why the rates observed in the West Midlands study were different from the rates we observed. However, these area and time period specific rates are based on small numbers of cases. The validation of cases in the GPRD can not necessarily be assumed to apply to all geographical areas. A recent study in North London estimated the prevalence of autism by year of birth and concluded there was a rise from 1979 to 1992 after which there was a plateau between 1992 and 1996 [27]. These authors excluded children with Asperger's. The review of clinical records of children in our study indicated that some children who would have been diagnosed as having autism in the early 1990s actually had Asperger's and would probably not have been classified as 'autism' in later years. This shift in diagnostic labelling from autism to Asperger's may explain, at least in part, the plateau in incidence described by Lingam et al. and would be compatible with the reduction in average age at diagnosis observed in that study.

## Conclusions

This is one of the largest studies undertaken of trends in the incidence of autism and other PDDs. We describe striking increases in the rates of diagnosis of these conditions. However, much of the increase may be due to better ascertainment related to changes in diagnostic practice and improved recognition of the conditions. While review of clinical records confirmed that over 90% of diagnoses of PDDs recorded by general practitioners were likely to be correct, the nature of the study precluded us from assessing how often children with PDDs were not diagnosed. Thus the extent to which the increases in incidence we document are true increases in disease is uncertain.

## Abbreviations

*PDD *pervasive developmental disorder

*GPRD *General Practice Research Database

## Competing interests

The authors declare that they have no competing interests.

## Authors' contributions

AH, LS, EF, LR and PS designed the study. LH and EF undertook the validation of case reports. CC analysed the data. LS drafted the paper. All authors commented on drafts and approved the final version of the paper.

## Pre-publication history

The pre-publication history for this paper can be accessed here:



## Supplementary Material

Additional File 1 Appendix: Codes used to identify cases, numbers identified, and diagnostic classification used in this paper Click here for file

## Figures and Tables

**Table 1:** First diagnoses of PDD by year of diagnosis and gender

**Year**	**Number of cases**			**Person years at risk**			**Crude incidence rate (per 10000 person-years)**			**Male:Female incidence rate ratio**	**Age standardised incidence ratio (S.I.R)**		
	**All**	**M**	**F**	**All**	**M**	**F**	**All**	**M**	**F**		**All**	**M**	**F**

**1988**	1	1	0	94474	49353	45121	0.11	0.20	0	∞	8	10	0
**1989**	6	6	0	260978	135542	125436	0.23	0.44	0	∞	19	22	0
**1990**	21	20	1	643830	332819	311012	0.33	0.60	0.03	18.7	28	32	8
**1991**	45	36	9	1118577	576722	541855	0.40	0.62	0.17	3.8	35	34	42
**1992**	45	37	8	1311605	675188	636417	0.34	0.55	0.13	4.3	31	31	33
**1993**	69	51	18	1376111	707674	668437	0.50	0.72	0.27	2.7	47	42	72
**1994**	77	61	16	1432528	735395	697134	0.54	0.83	0.23	3.6	52	49	64
**1995**	108	97	11	1476627	756200	720427	0.73	1.28	0.15	8.4	74	79	45
**1996**	102	85	17	1381242	705526	675716	0.74	1.21	0.25	4.8	78	78	77
**1997**	133	115	18	1283151	653791	629359	1.04	1.76	0.29	6.2	114	119	89
**1998**	192	152	40	1228058	624723	603335	1.56	2.43	0.66	3.7	180	173	218
**1999**	231	193	38	1121383	568937	552446	2.06	3.39	0.69	4.9	242	244	231
**2000**	211	179	32	935906	474344	461562	2.26	3.77	0.69	5.4	277	284	244
**2001**	169	139	30	567058	287574	279483	2.98	4.83	1.07	4.5	365	361	388
**All**	**1410**	**1172**	**238**	**14231526**	**7283787**	**6947739**	**0.99**	**1.61**	**0.34**	**4.8**	**100**	**100**	**100**

**Table 2:** Number of cases, person-years follow-up and rates of first recorded diagnosis of PDD (per 10,000 person-years) by year of diagnosis and year of birth (in two year categories). The diagonally linked numbers identify rates among children of the same age in different years

	**Year of first diagnosis**															
		
	**1992–93**			**1994–95**			**1996–97**			**1998–99**			**2000–01**		
	
**Birthyear**	n	PY	**rate**	n	PY	**rate**	n	PY	**rate**	n	PY	**rate**	n	PY	**rate**	
	
**73–74**	5	257638	**0.19**	5	263712	**0.19**	1	233838	**0.04**	4	194554	**0.21**	4	118997	**0.34**	age 26–28
**75–76**	0	233164	**0.00**	4	232393	**0.17**	5	208111	**0.24**	4	175331	**0.23**	2	107426	**0.19**	age 24–26
**77–78**	5	233093	**0.21**	2	227191	**0.09**	7	191785	**0.36**	8	162057	**0.49**	0	100419	**0.00**	age 22–24
**79–80**	1	265530	**0.04**	2	257752	**0.08**	9	211888	**0.42**	13	171491	**0.76**	3	104136	**0.29**	age 20–22
**81–82**	6	268477	**0.22**	3	262006	**0.11**	6	217307	**0.28**	20	174989	**1.14**	10	104093	**0.96**	age 18–20
**83–84**	10	275918	**0.36**	9	270128	**0.33**	4	226897	**0.18**	12	184013	**0.65**	5	110565	**0.45**	age 16–18
**85–86**	14	283299	**0.49**	11	277108	**0.40**	21	231591	**0.91**	28	187229	**1.50**	12	112080	**1.07**	age 14–16
**87–88**	26	294006	**0.88**	18	285548	**0.63**	16	237945	**0.67**	32	191788	**1.67**	31	114057	**2.72**	age 12–14
**89–90**	42	298297	**1.41**	45	289890	**1.55**	30	241271	**1.24**	48	194806	**2.46**	41	116246	**3.53**	age 10–12
**91–92**	5	248952	**0.2**	77	285511	**2.70**	53	238318	**2.22**	56	192373	**2.91**	51	114277	**4.46**	age 8–10
**93–94**	0	29342		9	231555	**0.39**	70	227807	**3.07**	89	184757	**4.82**	67	110641	**6.06**	age 6–8
**95–96**				0	26360		13	177284	**0.73**	98	176267	**5.56**	69	106292	**6.49**	age 4–6
**97–98**							0	20351		11	144307	**0.76**	81	104606	**7.74**	age 2–4
**99–00**										0	15479		4	79129	**0.51**	age 0–2

**Table 3:** Crude rates of first diagnosis (per 10 000 person-years) and age standardised incidence ratios for autism and other PDDs by year of diagnosis

**year**	**Total cases**	**Cases with 'other PDD' diagnosis**	**% cases with 'other PDD' diagnosis**	**incidence other PDDs**	**incidence autism**	**SIR other PDDs**	**SIR autism**
**1988**	1	0	0	0	0.11	0	10
**1989**	6	0	0	0	0.23	0	23
**1990**	21	0	0	0	0.33	0	35
**1991**	45	0	0	0	0.40	0	44
**1992**	45	0	0	0	0.34	0	39
**1993**	69	2	3	0.01	0.49	6	58
**1994**	77	2	3	0.01	0.52	6	64
**1995**	108	3	3	0.02	0.71	9	91
**1996**	102	8	8	0.06	0.68	27	92
**1997**	133	18	14	0.14	0.90	67	128
**1998**	192	51	27	0.42	1.15	204	173
**1999**	231	87	38	0.78	1.28	369	200
**2000**	211	82	39	0.88	1.38	435	225
**2001**	169	60	36	1.06	1.92	495	319

**Table 4:** Crude incidence rates (per 10 000 person-years) and age standardised incidence ratios (SIR) for the period 1988 to 2001 by geographical area of the United Kingdom (listed in order of SIR)

	**Autism**			**Other PDDs**			**ALL**		
**Region**	**Number of cases**	**Incidence rate**	**SIR**	**Number of cases**	**Incidence rate**	**SIR**	**Number of cases**	**Incidence rate**	**SIR (95%CI)**

WALES	40	0.64	83	1	0.02	7	41	0.65	66 (49,90)
NORTH WEST	121	0.60	78	32	0.16	72	153	0.76	77 (66,90)
NORTHERN & YORKSHIRE	86	0.74	98	11	0.10	43	97	0.84	85 (70,104)
N. IRELAND	29	0.71	97	7	0.17	79	36	0.88	93 (67,129)
EASTERN	132	0.76	97	35	0.20	90	167	0.96	95 (82,111)
SCOTLAND	32	0.62	84	15	0.29	135	47	0.91	96 (72,128)
LONDON	112	0.79	103	24	0.17	78	136	0.96	97 (82,115)
TRENT	94	0.79	100	24	0.20	90	118	0.99	98 (82,117)
SOUTH WEST	99	0.72	92	44	0.32	144	143	1.03	103 (87,121)
WEST MIDLANDS	146	0.85	113	36	0.21	97	182	1.06	109 (94,126)
SOUTH EAST	206	1.00	128	84	0.41	184	290	1.40	141 (126,158)
