# Supplementary material for: Rate of first recorded diagnosis of autism and other pervasive developmental disorders in United Kingdom general practice, 1988 to 2001
Source: BMC Med. 2004 Nov 9;2:39. doi: 10.1186/1741-7015-2-39 (PMC533883; doi:10.1186/1741-7015-2-39)
Supplement: Additional File 1 — Appendix: Codes used to identify cases, numbers identified, and diagnostic classification used in this paper [file 1741-7015-2-39-S1.doc]

| **Code description** | **Oxmis/Read** | **Code** | **No. cases identified**  **(N=1410)** | **Autism/ other PDD** |
| --- | --- | --- | --- | --- |
| autism | O | 2958B | 428 | autism |
| autism | R | E140.12 | 318 | autism |
| Asperger's syndrome | R | Eu84500 | 294 | other PDD |
| autistic child | O | 2958BA | 155 | autism |
| autistic disorder | R | Eu84011 | 154 | autism |
| infantile autism | R | E140.00 | 22 | autism |
| childhood autism | R | Eu84000 | 13 | autism |
| pervasive developmental disorders | R | Eu84.00 | 9 | other PDD |
| atypical autism | R | Eu84100 | 6 | other PDD |
| childhood autism | R | E140.13 | 5 | autism |
| active infantile autism | R | E140000 | 2 | autism |
| atypical childhood psychosis | R | Eu84111 | 2 | other PDD |
| pervasive developmental disorder, unspecified | R | Eu84z00 | 2 | other PDD |
| kanner's syndrome | R | E140.11 | 0 | autism |
| residual infantile autism | R | E140100 | 0 | autism |
| infantile autism nos | R | E140z00 | 0 | autism |
| disintegrative psychosis | R | E141.00 | 0 | autism |
| heller's syndrome | R | E141.11 | 0 | autism |
| active disintegrative psychoses | R | E141000 | 0 | autism |
| residual disintegrative psychoses | R | E141100 | 0 | autism |
| disintegrative psychosis nos | R | E141z00 | 0 | autism |
| infantile autism | R | Eu84012 | 0 | autism |
| infantile psychosis | R | Eu84013 | 0 | autism |
| kanner's syndrome | R | Eu84014 | 0 | autism |
| mental retardation with autistic features | R | Eu84112 | 0 | autism |
| other childhood disintegrative disorder | R | Eu84300 | 0 | autism |
| dementia infantalis | R | Eu84311 | 0 | autism |
| disintegrative psychosis | R | Eu84312 | 0 | autism |
| heller's syndrome | R | Eu84313 | 0 | autism |
| symbiotic psychosis | R | Eu84314 | 0 | other PDD |
| overactive disorder assoc mental retard/stereotype movts | R | Eu84400 | 0 | autism |
| autistic psychopathy | R | Eu84511 | 0 | other PDD |
| other pervasive developmental disorders | R | Eu84y00 | 0 | other PDD |

**Appendix: Codes used to identify cases, numbers identified, and diagnostic classification used in this paper**
